# Metabolic Remodeling and Mitochondrial Stress in Atrial Fibrillation: Mechanisms and Translational Targets

**DOI:** 10.31083/RCM44688

**Published:** 2025-12-18

**Authors:** Konstantinos Grigoriou, Paschalis Karakasis, Panagiotis Theofilis, Panayiotis K Vlachakis, Nikias Milaras, Dimitrios Patoulias, Antonios P Antoniadis, Nikolaos Fragakis

**Affiliations:** ^1^Department of Pharmacology, School of Medicine, National and Kapodistrian University of Athens, 15771 Athens, Greece; ^2^Second Department of Cardiology, Hippokration General Hospital, Aristotle University of Thessaloniki, 54642 Thessaloniki, Greece; ^3^First Cardiology Department, School of Medicine, Hippokration General Hospital, National and Kapodistrian University of Athens, 11527 Athens, Greece; ^4^Second Propedeutic Department of Internal Medicine, Faculty of Medicine, School of Health Sciences, Aristotle University of Thessaloniki, 54124 Thessaloniki, Greece

**Keywords:** atrial fibrillation, metabolic remodeling, mitochondrial stress, SGLT2 inhibitors, GLP-1 receptor agonists

## Abstract

Atrial fibrillation (AF) is the most prevalent cardiac arrhythmia and frequently co-occurs with metabolic diseases, such as diabetes and obesity. Due to the intricate and multifactorial pathophysiology of AF, this disorder often eludes effective prevention and durable control with current therapeutic strategies; thus, these strategies may not consistently mitigate the onset, persistence, and related adverse outcomes of AF. Moreover, atrial metabolic remodeling and mitochondrial stress can promote the development of atrial cardiomyopathy and AF through electrophysiological and structural changes. Hence, targeting these metabolic alterations may prevent the onset of this arrhythmia. A contemporary therapeutic paradigm prioritizes restoration of metabolic homeostasis, led by sodium–glucose cotransporter 2 (SGLT2) inhibitors and glucagon-like peptide-1 (GLP-1) receptor agonists and complemented by emerging mitochondria-targeted strategies with potential for incremental disease modification. Concurrently, integrative multi-omics is mapping atrial metabolic diversity in AF to support biomarker-guided, individualized interventions, while next-generation imaging is enhancing the detection of pathologic substrates and refining risk assessment. This review provides a comprehensive analysis of the mechanisms through which metabolic remodeling and mitochondrial stress cause AF, evaluates current experimental and diagnostic methods, and discusses emerging substrate-targeted therapies.

## 1. Introduction

Atrial fibrillation (AF) is the most prevalent cardiac arrhythmia among adults and a significant contributor to global morbidity and mortality [1, 2]. The incidence and prevalence of AF have shown a significant rise, partially as a result of the aging population and improved survival rates from chronic diseases [2]. Globally, an estimated 59 million individuals are affected by AF, with a lifetime risk of one in three to five for those aged 45 and older [3]. The development of AF is affected by a range of modifiable and non-modifiable risk factors. Among them, cardiometabolic diseases, such as diabetes and obesity, are increasingly recognized as key components of AF pathogenesis [4, 5, 6, 7, 8]. Hence, treatment strategies targeting metabolic pathways may offer disease-modifying benefits.

**Fig. 1.**
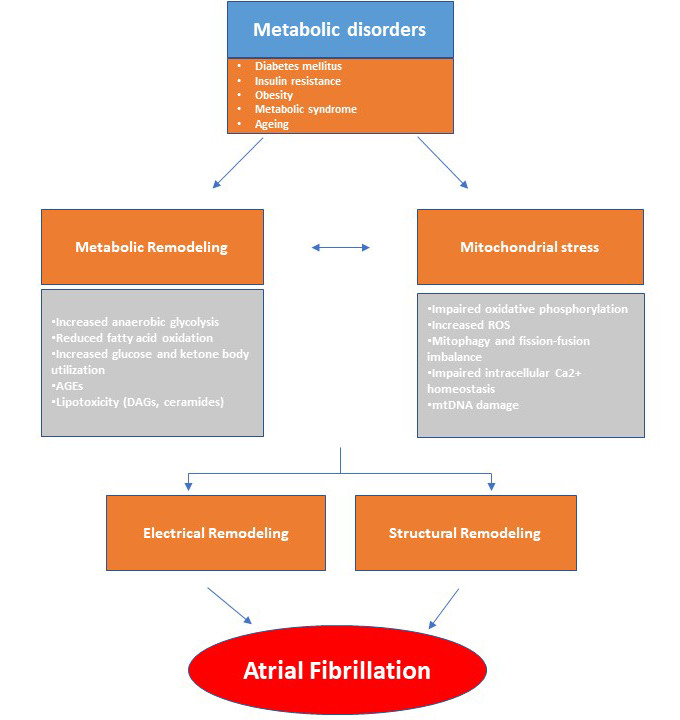
**This diagram illustrates how metabolic remodeling and mitochondrial stress play a pivotal role in the development of atrial fibrillation (AF), arising from systemic metabolic disorders**. In response to metabolic stress, the atrial myocardium undergoes a metabolic shift characterized by alterations in lipid, glucose, and ketone metabolism. These changes, along with mitochondrial dysfunction, contribute to a pro-arrhythmic environment. Metabolic disturbances, in conjunction with structural and electrical remodeling, promote the abnormal electrical activity characteristic of AF. AGEs, advanced glycation end-products; DAGs, diacylglycerols; ROS, reactive oxygen species; mtDNA, mitochondrial DNA.

Current treatments often fail to prevent AF and its complications [9]. Historically considered a purely electrical disease, AF is now growingly recognized as the clinical manifestation of a complex and multifaceted entity (atrial cardiomyopathy), characterized by structural, electrical and molecular changes of the atrial myocardium [10]. Structural remodeling refers to atrial enlargement, tissue fibrosis, and reduced contractility. Electrical remodeling on the other hand, consists of ion channel dysfunction and conduction abnormalities within the atria [11, 12]. There is a growing body of research that underscores the fundamental changes in atrial metabolism and energy homeostasis, which often precede the onset of AF. This metabolic remodeling encompasses a diverse range of alterations within the atria to address the heightened metabolic demands of the arrhythmia. A central component of this process is mitochondrial stress, which sustains the altered metabolic state [8]. Over time, these changes can become maladaptive and enhance atrial arrhythmogenic processes [13, 14] (Fig. 1).

This review synthesizes current mechanistic insights into the concept of metabolic remodeling and delineates the central role of mitochondrial stress in the pathogenesis of AF. Additionally, emerging therapeutic approaches targeting the metabolic substrate of AF are reviewed, current imaging techniques for quantifying metabolic substrate within the atria are evaluated, and the utility of multi-omics and experimental animal models is explored. Finally, we discuss existing knowledge gaps and propose future research directions to holistically address AF.

## 2. Methods 

A comprehensive literature search was conducted using PubMed, Scopus, and Web of Science databases up to July 2025. The search strategy combined controlled vocabulary and free-text terms, including but not limited to atrial fibrillation, metabolic remodeling, mitochondrial stress, atrial cardiomyopathy, electrical remodeling, structural remodeling, atrial remodeling, atrial fibrosis, fusion, fission, mitochondrial biogenesis, glucagon-like peptide-1 (GLP-1) receptor agonists, and sodium-glucose cotransporter 2 (SGLT2) inhibitors. We screened original experimental studies, clinical trials, meta-analyses, and high-quality mechanistic reviews published in English for relevance to the scope of this study. Additional references were identified through manual citation tracking of pertinent articles.

## 3. Mitochondrial Function in Cardiomyocyte Homeostasis

In order for the myocardium to sustain its normal contraction, it needs a continuous supply of energy, primarily generated through mitochondrial oxidative phosphorylation [15, 16, 17]. During this process, various available substrates are converted into adenosine triphosphate (ATP), and their availability depends on several factors such as oxygen (O_2_) supply, nutrient efficiency, and workload [16]. Under physiologic conditions, fatty acids are the main substrate, accounting for up to 90% of the myocardium’s energy supply [18]. After their β-oxidation, fatty acids converge into acetyl coenzyme A (acetyl CoA), which enters the tricarboxylic acid (TCA) cycle. This flux generates the reducing equivalents nicotinamide adenine dinucleotide (NADH) and flavin adenine dinucleotide (FADH_2_), which feed electrons into the respiratory chain via complexes I and II, respectively. The electron transport chain (ETC), in turn, drives protons into the intermembrane space, generating ATP, a process which requires O_2_. Another source of ATP is pyruvate derived from glucose, lactate, and ketone bodies. Their role in the energy supply of the normal myocardium is limited but may be upregulated in cardiac diseases [16, 18].

In addition, mitochondria are the primary intracellular source of reactive oxygen species (ROS). During oxidative phosphorylation, a small portion of electrons derived from NADH and FADH_2_ (approximately 0.2–2%) escape the ETC and react aberrantly with O_2_, resulting in the formation of superoxide (O_2_^–^) [18]. At controlled levels, ROS are involved in numerous cellular signaling pathways that regulate cell growth, adhesion, differentiation, and apoptosis. However, excessive ROS accumulation can damage to DNA, proteins, and lipids [19, 20, 21]. To counteract these effects, cells are equipped with an efficient antioxidant defense system. This system encompasses numerous enzymes, such as manganese superoxide dismutase (Mn-SOD), mitochondrial glutaredoxin reductase, and glutathione peroxidase 4 [22].

Moreover, mitochondria regulate intracellular calcium (Ca^2+^) signaling pathways and serve as a reservoir for Ca^2+^ [23]. Mitochondrial Ca^2+^ homeostasis depends on the proper uptake and release of Ca^2+^. Ca^2+^ uptake is primarily regulated by the mitochondrial Ca^2+^ uniporter (MCU) complex located on the inner mitochondrial membrane, while Ca^2+^ release is mediated by the sodium-calcium exchanger (NCX) and the mitochondrial permeability transition pore (mPTP) [24]. Dysregulation of mitochondrial Ca^2+^ increases oxidative stress impairs cellular metabolism and contributes to atrial remodeling, thereby promoting an arrhythmogenic substrate [25, 26].

Of note, mitochondrial distribution and density are significantly lower in the atria compared to the ventricles, a difference that may influence their Ca^2+^ signaling dynamics [27, 28, 29]. Tanaami *et al*. [29] demonstrated that atrial myocytes exhibit impaired local control of Ca^2+^ release, with propagation following Ca^2+^ release induced by stimulation, compared to ventricular myocytes. In addition, the time between the peak of the Ca^2+^ transient and peak contraction was shorter in atrial myocytes.

## 4. Metabolic Remodeling in Atrial Fibrillation: From Substrate to Trigger

### 4.1 Energy Substrate Shifts: From Oxidative Phosphorylation to Glycolysis

AF is increasingly recognized as a metabolic cardiac disease, characterized by high energy demand and features resembling ischemia [26]. To address these needs, atrial O_2_ supply and consumption increase two- to three-fold following acute AF induction [30, 31]. At the same time, reductions in high-energy phosphate compounds, including ATP and creatine phosphate, along with decreased activity of phosphotransfer enzymes, are observed [26].

As previously mentioned, in cardiac diseases, energy substrates other than fatty acids are utilized to a greater extent to generate ATP in cardiomyocytes [16, 18, 32]. In AF, the atrial myocardium undergoes notable metabolic shifts, characterized by increased anaerobic glycolysis, decreased glucose and fatty acid oxidation, and enhanced reliance on ketone body metabolism [26]. This metabolic change, especially in persistent AF, provides a more efficient energy source and possesses cardioprotective properties by reducing oxidative stress and inflammation. However, the long-term effects of chronic ketosis and its role in AF progression and treatment remain to be determined [26].

In AF, glucose metabolism shifts toward a fetal-like state, characterized by increased glucose uptake and enhanced glycolytic activity [33]. This process involves dysregulation of key glycolytic enzymes and glucose transporters such as glucose transporter (GLUT) 1 and GLUT4, while pyruvate dehydrogenase (PDH) activity is decreased due to increased expression of its inhibitor, PDH kinase (PDK) [33, 34]. The resulting uncoupling of glycolysis from oxidative metabolism reflects a Warburg-like metabolic phenotype [34]. Additionally, altered fatty acid metabolism, marked by lipid accumulation and impaired fatty acid oxidation, contributes to arrhythmogenesis [35, 36]. Key molecular pathways involved include sirtuin 3 (SIRT3)/AMP-activated protein kinase (AMPK) signaling, carnitine palmitoyltransferase-1, acetyl-CoA carboxylase (ACC), and peroxisome proliferator-activated receptor α (PPARα) [37, 38, 39].

### 4.2 AMPK, SIRT1, and Mitochondrial Biogenesis

Mitochondrial biogenesis is a multifaceted process by which cells increase mitochondrial mass and number to expand their energy expenditure. It involves dynamic events such as mitochondrial fission and fusion, and is triggered by environmental and physiological stimuli, such as exercise, fasting, oxidative stress, proliferation, and differentiation [40, 41, 42, 43].

Peroxisome proliferator-activated receptor gamma coactivator 1-alpha (PGC-1α), encoded by the *PPARGC1A* gene, plays a pivotal role in mitochondrial biogenesis. It upregulates nuclear-encoded mitochondrial proteins and activates nuclear respiratory factor 1 (NRF-1) and nuclear factor erythroid 2-related factor 2 (NRF-2), which in turn regulate mitochondrial genes, including mitochondrial transcription factor A (*TFAM*) and mitochondrial RNA polymerase (*POLRMT*) [44, 45].

PGC-1α expression is tuned by AMPK and sirtuin 1 (SIRT1) via phosphorylation and deacetylation, respectively [40]. AMPK acts as an energy sensor of the cell and becomes activated when the AMP/ATP ratio is elevated. Activated AMPK enhances catabolism, increases mitochondrial and glucose metabolism, and inhibits lipid synthesis [46]. SIRT1, using NAD^+^ as a substrate, activates PGC-1α and modulates additional transcription factors such as peroxisome proliferator-activated receptor gamma (PPARγ), p53, and the forkhead box O (FOXO) family [46, 47, 48, 49].

A considerable body of evidence suggests that both AMPK and SIRT1 are involved in the pathophysiological mechanisms of AF. A recent study demonstrated that SIRT1 effectively attenuates age-related AF by suppressing atrial necroptosis through regulation of receptor-interacting protein kinase 1 (RIPK1) acetylation [50]. Another study established a correlation between SIRT1 levels and the development of AF following cardiac surgery [51]. Notably, SIRT1 has also been associated with increased expression of collagen I in left atrial tissue in patients with mitral regurgitation (MR), providing novel insights and potential strategies for diagnosing and treating atrial fibrosis [52].

Emerging evidence has implicated AMPK as a pivotal mediator against the development of atrial remodeling and the promotion of AF. Indeed, in atrial cardiomyocytes subjected to AF-related metabolic stress, AMPK activation preserved cell contractility and Ca^2+^ homeostasis [53]. In mice models, atria-specific deletion of AMPK and *Lkb1*—which encodes liver kinase B1 (LKB1), a primary upstream activator of AMPK—resulted in electrical and structural remodeling of the atria, further underscoring its anti-AF significance [54, 55, 56]. Similarly, reduced activation of AMPK was noted in a mouse model of cardiometabolic heart failure with preserved ejection fraction (HFpEF), which had increased vulnerability to pacing-induced AF. Administration of metformin, an AMPK activator, reduced AF susceptibility in these mice, highlighting the pivotal role of AMPK in AF associated with metabolic disorders [57].

### 4.3 Redox Imbalance and Reactive Oxygen Species

Oxidative stress defines a state in which ROS generation overrides cellular antioxidant defense mechanisms. This process is mediated by several enzymes, including nicotinamide adenine dinucleotide phosphate (NADPH) oxidase (NOX), xanthine oxidase, and uncoupled nitric oxide (NO) synthase (NOS) [58]. Aberrant ROS accumulation impairs redox signaling and contributes to the pathogenesis of various cardiovascular diseases, including cardiac rhythm disorders [59, 60, 61, 62, 63].

ROS are a class of oxygen-containing molecules, including peroxynitrite (ONOO⁻), superoxide (O_2_⁻), hydroxyl radicals (-OH), and hydrogen peroxide (H_2_O_2_). There are several possible ways for ROS to form an atrial arrhythmogenic substrate. These reactive species activate inflammatory pathways, induce ion channel remodeling, dysregulate intracellular Ca^2+^ homeostasis, reduce NO availability, and enhance atrial fibrosis [62, 64, 65, 66]. Of note, the effect of oxidative stress on AF may depend on the source of ROS, the specific cellular microdomains where they exert their effects, and NO levels [13].

The human body has a combined antioxidant system, which includes enzymatic and non-enzymatic antioxidants to counteract the negative effects of ROS. The first line of defense is the group of enzymatic antioxidants, including superoxide dismutase (SOD), catalase (CAT), and glutathione peroxidase (GPx). The second line consists of non-enzymatic scavenging antioxidants, which directly neutralize free radicals, such as ascorbic acid, alpha-tocopherol, uric acid (UA), and glutathione. Evidence from many investigations has established an association between biomarkers of oxidative stress, such as total antioxidant capacity and ROS levels, and the presence of arrhythmias [67, 68, 69, 70].

### 4.4 Fatty Acid Oxidation and Lipotoxicity in Atrial Tissue 

Lipotoxicity has emerged as a dynamic and important contributor to the pathogenesis of atrial cardiomyopathy and AF [13]. The associations between lipotoxicity and metabolic diseases such as diabetes mellitus and obesity are well established. Under these conditions, fatty acid uptake often outweighs their catabolism and leads to the excess accumulation of toxic lipid intermediates, which cause direct damage to cardiomyocytes [13, 71].

Ceramides and diacylglycerols (DAGs) are among the best-studied lipid intermediates involved in lipotoxicity [71]. These molecules are thought to play a role in arrhythmogenesis, particularly by triggering several molecular pathways, including inflammation, apoptosis, and insulin resistance [13]. For example, DAGs can activate protein kinase C (PKC), which in turn triggers nuclear factor kappa B (NF-κB) and enhances the release of pro-inflammatory cytokines, like tumor necrosis factor (TNF)—a key player in the development of atrial AF [72, 73, 74, 75].

Despite these findings, the exact role of lipotoxicity in the pathogenesis of AF remains incompletely understood. Lipid accumulation has been observed in the atrial tissue of patients with AF [76], and dysfunction in fatty acid transport and mitochondrial oxidation has been linked to atrial arrhythmogenesis [35, 77, 78]. Pediatric cases involving deficiencies in enzymes essential for mitochondrial fatty acid entry—such as carnitine palmitoyltransferase II and carnitine-acylcarnitine translocase—have been associated with atrial conduction abnormalities and tachyarrhythmias [77]. *In vitro* studies have also demonstrated that exposing atrial cardiomyocytes to fatty acids results in the shortening of action potential duration (APD), increased repolarizing potassium currents (I_K_), and a higher incidence of delayed afterdepolarizations (DADs) [78].

While these atrial-specific findings offer promising insights, the current understanding of cardiac lipotoxicity is predominantly derived from studies focused on the ventricles. Targeted research is urgently needed to determine whether similar lipotoxic mechanisms underlie atrial cardiomyopathy in the setting of metabolic diseases.

## 5. Mitochondrial Dysfunction in Atrial Fibrillation

### 5.1 Mitochondrial DNA Damage and Depletion

Mitochondria harbor a distinct circular, double-stranded genome—mitochondrial DNA (mtDNA)—which encodes a subset of genes essential for metabolic regulation, calcium signaling, redox balance, and the modulation of apoptosis and mitophagic processes [79]. MtDNA is more vulnerable to oxidative damage compared to nuclear DNA owing to its proximity to the primary site of ROS generation and the absence of histones [80]. These factors may compromise the stability of mitochondrial genome, contributing to the pathogenesis of various diseases, including AF [81].

ROS cause direct oxidative damage to mtDNA via modification of bases, such as the frequent formation of 8-oxo-7,8-dihydroguanine (8-oxoG) [82]. ROS also oxidize DNA polymerase gamma (POLG), the enzyme responsible for mtDNA replication and repair, thereby indirectly impairing these processes [83]. There is a bidirectional relationship between oxidative stress and mtDNA damage; while ROS cause mtDNA damage, the resulting mitochondrial dysfunction amplifies ROS generation, creating a self-perpetuating cycle of oxidative injury [58, 81, 84].

Deletions of mtDNA have been reported in AF patients; however, it remains unclear whether these deletions are a cause or a consequence of impaired ATP synthesis, aging, hemodynamic compromise, or AF itself [85, 86]. Additionally, reduced mtDNA copy number—a marker of mitochondrial dysfunction—has been linked to a higher risk of AF, independent of traditional cardiovascular risk factors [87].

Mitochondrial quality control mechanisms—such as fission, fusion, and mitophagy—are essential for removing damaged mitochondria and preserving cellular homeostasis [88]. When these processes are dysregulated, mtDNA can be released into the cytoplasm or extracellular space, where it acts as damage-associated molecular pattern (DAMP) [89]. DAMPs aggravate the inflammatory response via activation of the cyclic GMP–AMP synthase–stimulator of interferon genes–TANK-binding kinase 1 (cGAS–STING–TBK1) pathway in immune cells, leading to cytokine production through interferon regulatory factor 3 (IRF3) and NF-κB signaling, and promoting activation of the NOD-like receptor protein 3 (NLRP3) inflammasome—an established contributor to arrhythmogenesis. Toll-like receptor 9 (TLR9) may also mediate these inflammatory responses [90, 91, 92]. Elevated circulating mtDNA levels have been identified in human atrial tissue from patients with diverse AF phenotypes, including postoperative and paroxysmal forms, suggesting its value as a potential biomarker [93, 94, 95].

### 5.2 Impaired Mitophagy and Fission-Fusion Imbalance

**Fig. 2.**
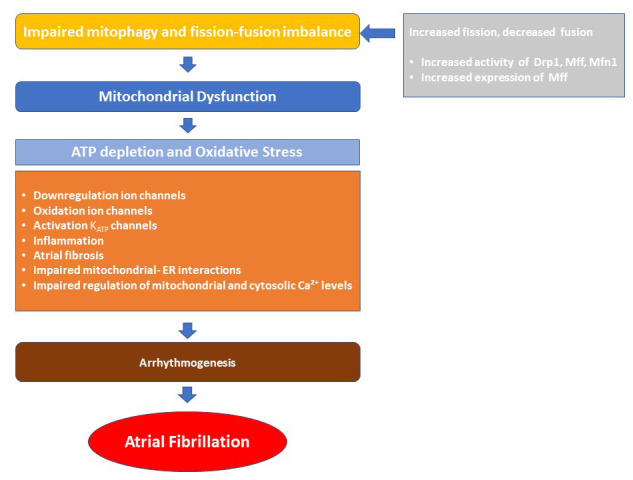
**Pathophysiological drivers of mitochondrial dysfunction in atrial fibrillation**. Mitochondrial dynamics—including fission, fusion, and mitophagy—are essential for maintaining mitochondrial function and cellular metabolism. An imbalance in these processes can lead to mitochondrial dysfunction, characterized by impaired energy production and increased oxidative stress. These alterations contribute to atrial arrhythmogenesis through mechanisms such as ion channel remodeling, inflammation, atrial fibrosis, disrupted mitochondrial–ER interactions, and impaired regulation of mitochondrial and cytosolic Ca^2+^ levels. Drp1, dynamin-related protein 1; Mff, mitochondrial fission factor; Mfn1, mitofusion-1; ATP, adenosine triphosphate; K_ATP_, ATP-sensitive potassium channel; ER, endoplasmic reticulum; Ca^2+^, calcium.

Mitophagy is the selective elimination of damaged mitochondria through autophagy to maintain cellular homeostasis (Fig. 2). Mitophagic dysfunction has been observed in several cardiovascular disorders, such as atherosclerosis and cardiomyopathy. Accumulating evidence also suggests its involvement in the pathogenesis of AF [96, 97]. Indeed, dysregulated mitophagy causes mitochondrial dysfunction, which in turn leads to ATP depletion and excessive ROS generation. This promotes atrial ion channel dysregulation and mtDNA damage, alters gene transcription, and activates inflammatory signaling [98, 99]. Furthermore, oxidative stress enhances the oxidation of ryanodine receptors (RyR) and, Ca^2+^/calmodulin-dependent protein kinase II (CaMKII), and contributes to atrial fibrosis [100]. Therefore, it can be reasonably speculated that mitophagy, by eliminating damaged mitochondria and regulating ROS and ATP levels, may inhibit or slow the progression of AF.

Mitochondria have been reported to be structurally abnormal in the atrial tissue of patients with chronic AF [97]. Markers of impaired autophagic flux were evident, such as reduced levels of microtubule-associated protein 1 light chain 3 beta (LC3B) II, a decreased LC3B II/I ratio, elevated p62, and increased expression of cytochrome c oxidase IV (Cox IV). Furthermore, mitochondrial engulfment by autophagosomes was disrupted [97]. In a recent study, a novel correlation was found between autophagy and the recurrence of AF following catheter, ablation using metabolomic analysis. Notably, lower serum levels of Parkin, a representative biomarker of mitophagy, predicted AF recurrence of very late onset. These findings underscore the need for further research to elucidate the mechanisms by which mitochondrial autophagy contributes to the pathogenesis of AF [101].

Mitochondrial fission refers to the dynamic remodeling event in which a singular mitochondrial organelle undergoes division, generating two or more discrete mitochondria. This process is primarily regulated by the GTPase dynamin-related protein 1 (Drp1), which is recruited to the mitochondrial outer membrane (OMM) by adaptor proteins such as fission 1 protein (Fis1), mitochondrial fission factor (Mff), and mitochondrial dynamics proteins of 49 and 51 kDa (MiD49/51) [100]. Conversely, mitochondrial fusion is a process by which two or more mitochondria merge to form a larger organelle. This phenomenon is coordinated by the outer membrane GTPases mitofusin 1 and 2 (Mfn1/2), which mediates fusion of the OMM, and by the inner membrane protein optic atrophy 1 (Opa1), which enables fusion of the inner mitochondrial membrane [102]. An imbalance between mitochondrial fission and fusion, along with impaired mitophagy, may lead to a proarrhythmic atrial substrate through mitochondrial oxidative stress, reduced energy supply and impaired Ca^2+^ homeostasis [103].

### 5.3 Calcium Overload and Mitochondrial Permeability Transition Pore Opening

Mitochondrial Ca^2+^ is essential for cellular energy production. It directly activates enzymes in the TCA cycle, supports the ETC, and boosts ATP production. It also preserves redox homeostasis by supporting antioxidant mechanisms, thereby mitigating the adverse effects of ROS [25].

Changes in Ca^2+^ cycling can occur within days following rapid atrial stimulation, initially presenting as a form of Ca^2+^ signaling silencing [104]. This early response serves a cardioprotective role but eventually becomes maladaptive as paroxysmal AF develops and progresses to a permanent form. This transition occurs due to enhanced diastolic Ca^2+^ leak from RyRs, which contributes to the genesis of afterdepolarizations. Higher intracellular Ca^2+^ levels also trigger pro-fibrotic signaling pathways, leading to structural remodeling [104]. These changes create a substrate conducive to the complex re-entry circuits that sustain AF.

Moreover, Ca^2+^ overload results in increased ROS production, mPTP opening, and oxidative damage. This cascade leads to mitochondrial dysfunction, further Ca^2+^ leakage, and cardiomyocyte apoptosis, all of which contribute to atrial remodeling and AF progression [104, 105].

There are two main mechanisms by which mitochondrial Ca^2+^ is released. One involves the sodium-calcium exchanger (NCLX), and the other is governed by the mPTP [104]. Under stress conditions, such as Ca^2+^ overload, oxidative stress, inflammation, and energy depletion, transient openings of the mPTP may occur. This can lead to rapid Ca^2+^ release, loss of mitochondrial membrane potential, and cellular damage, including apoptosis or necrosis, and lipid peroxidation—all of which promote arrhythmogenesis [25, 106, 107, 108, 109].

### 5.4 Crosstalk With Endoplasmic Reticulum Stress

The endoplasmic reticulum (ER) is a vital organelle in eukaryotic cells, responsible for protein folding and secretion, Ca^2+^ regulation, and lipid metabolism [110]. When the ER’s capacity for protein folding becomes saturated, unfolded or misfolded proteins accumulate. This phenomenon disrupts ER function and leads to a condition known as ER stress [111, 112, 113]. Adaptive mechanisms such as ER-associated degradation (ERAD), the unfolded protein response (UPR), and reticulophagy, are then activated to restore ER function [110, 114].

Various cardiovascular and metabolic diseases can cause ER stress. The activated UPR is mediated by three ER-associated integral membrane proteins: protein kinase RNA-like ER kinase (PERK), inositol-requiring enzyme 1α (IRE1α), and activating transcription factor 6 (ATF6), which function to counteract ER stress. Dysfunction of the UPR may promote arrhythmogenesis through enhanced ROS production, inflammation, and impaired autophagy and apoptosis [115].

The ER also regulates Ca^2+^ homeostasis. When Ca^2+^ channels in the ER or plasma membrane open, cytosolic Ca^2+^ levels rise to support essential functions such as cardiac contraction and metabolism. The sarcoplasmic/ER Ca^2+^-ATPase 2a (SERCA2a) is responsible for cycling Ca^2+^ into the sarcoplasmic reticulum (SR) during heart muscle contraction and relaxation. Following ER stress, Ca^2+^ homeostasis is disrupted, leading to increased cytosolic Ca^2+^, which binds to calmodulin and activates multiple signaling pathways, such as calcium/calmodulin-dependent protein kinase kinase 2 (CaMKK2) and calmodulin–NADPH oxidase 2 (NOX2)–ROS–NRF2, which may promote arrhythmogenesis [115, 116].

Last, efficient ER–mitochondria communication is essential for cellular functions such as Ca^2+^ and lipid exchange, iron homeostasis, and immune regulation [117]. This crosstalk occurs at mitochondria-associated membranes (MAMs), which facilitate Ca^2+^ transfer, support energy production, regulate redox balance, and modulate apoptosis and ER stress [118]. Disruption of this interaction may contribute to AF development.

## 6. Integration With Electrophysiological Remodeling

Electrical remodeling refers to any change in the electrophysiological properties of the atrial myocardium that facilitates the genesis and progression of AF. One of the major aspects of this process is the reduction of APD, which, in turn, shortens the atrial effective refractory period (ERP). Reduced conduction velocity has also been shown to be part of this process [13, 119]. These electrophysiological alterations are mainly mediated by impaired ion channel function [120, 121]. In the context of metabolic remodeling, disrupted metabolic homeostasis and excessive ROS production further exacerbate these ion dynamic changes, thereby promoting and sustaining AF [13, 26, 122, 123].

Numerous ionic currents have been demonstrated to be redox-sensitive, including the L-type Ca^2+^ current (I_Ca,L_), inward-rectifier potassium current (I_K1_), ultra-rapid delayed rectifier potassium current (I_Kur_), and, more recently, the acetylcholine-activated potassium current (I_K,ACh_) [64]. In patients with chronic AF undergoing cardiac surgery, increased nitrosylation of the α1c subunit of L-type Ca^2+^ channels was observed, while treatment with the ROS scavenger N-acetylcysteine enhanced I_Ca,L_ compared to controls [121]. However, another study failed to confirm an effect of ROS inhibition on I_Ca,L_, suggesting that the role of oxidative stress in regulating I_Ca,L_ and contributing to AF substrate formation remains unclear [64, 124].

Similarly, research on I_K1_ regulation by oxidative stress has produced conflicting findings: in atrial myocytes from non-AF cardiac surgery patients, S-nitrosylation of Kir2.1 increased channel open probability, whereas in a canine atrial tachypacing model of AF, inhibition of ROS from NOX2 or mitochondria had no effect on I_K1_ [124, 125].

Alterations in I_Kur_ mediated by Kv1.5 channels have been associated with AF, including both gain- and loss-of-function variants in the *KCNA5* gene [126, 127, 128]. However, the precise contribution of I_Kur_ to AF remains uncertain, as its inhibition yields variable effects on APD and arrhythmia susceptibility depending on the underlying atrial electrophysiological state [64].

The constitutively active component of I_K,ACh_ (I_KH_) is upregulated in AF despite reduced or unchanged expression of its underlying channel subunits, Kir3.1/3.4, due to slowed channel closure and a consequent increase in open probability [64, 129, 130, 131, 132]. Moreover, oxidative stress contributes to AF by promoting PKCϵ translocation, which enhances I_KH_ conductance through channel phosphorylation. Inhibition of NOX2-derived ROS blocks this pathway, preventing I_KH_ upregulation and the associated shortening of the APD [133].

ROS contribute to an arrhythmogenic substrate through multiple interconnected pathways, in addition to their effects on atrial ion channels. One important mechanism involves oxidative activation of RyR2 and CaMKII, which causes abnormal Ca^2+^ release from the SR. This effect is further amplified by c-Jun N-terminal kinase 2 (JNK2), a stress-responsive kinase, which enhances CaMKII activity and independently increases SERCA2 function [134, 135]. By upregulating fibrotic gene expression and fibroblast proliferation, persistent oxidative stress also activates the transforming growth factor β1 (TGFβ1)/Smad3 pathway, which promotes atrial fibrosis [136]. In addition, redox imbalance can trigger DADs, further promoting arrhythmogenesis [137].

A major hallmark in the maintenance of AF is the depletion of high-energy phosphate stores. ATP-sensitive potassium (K_ATP_) channels open in response to reduced ATP levels. This action facilitates energy conservation by shortening APD and reducing Ca^2+^ influx. However, this adaptive response concurrently increases electrical heterogeneity, thereby promoting conduction abnormalities within the atrial myocardium [138]. Furthermore, metabolic stress disrupts ATP-dependent pumps such as SERCA, plasma membrane Ca^2+^-ATPase (PMCA), and Na^+^/K^+^ ATPase (NKA), leading to imbalances in Ca^2+^ and sodium levels. This, combined with cyclical K_ATP_ channel activation and suppressed I_Ca,L_, worsens electrical instability in the atria, promoting arrhythmias linked to energy depletion [138].

In summary, dysregulation of energy flux and oxidative stress can impair ion homeostasis, contributing to electrical remodeling of the atria and creating a substrate for AF. The interplay between energy and ion flux dysregulation is complex and multifactorial, making treatment challenging.

## 7. Metabolic Diseases and Atrial Fibrillation

Metabolic dysregulation is one of the major hallmarks in the development of AF. Due to their high energy demands, the fibrillating atria require metabolic flexibility in order to adjust to stress and enable rapid energy production [138]. However, metabolic diseases such as diabetes and obesity hinder this ability by disrupting the function of key metabolic regulators and the availability of energy substrates, thereby promoting AF initiation and maintenance [26].

Multiple molecular mechanisms contribute to atrial remodeling in individuals with diabetes or obesity. One shared electrophysiological characteristic is the slowing of atrial conduction velocity, caused by disrupted connexin distribution and reduced I_Na,peak_ [139, 140]. In addition, atrial remodeling in both conditions is associated with more frequent arrhythmogenic Ca^2+^ release events from the SR during diastole. In obesity, this is largely driven by inflammasome activation, which is promoted by increased intestinal permeability and elevated circulating levels of lipopolysaccharides (LPS) [141]. In contrast, diabetes-related Ca^2+^ release has been attributed to activation of CaMKII by oxidative stress [101].

An important aspect of structural remodeling observed in both conditions is atrial fibrosis. Obesity alters the epicardial adipose tissue secretome, shifting it toward a profibrotic phenotype. In diabetes, the underlying mechanism involves profibrotic cytokine release from activated atrial fibroblasts. Together, these electrophysiological and structural changes create an atrial arrhythmogenic substrate [13].

Of note, metabolic syndrome—defined by elevated waist circumference, elevated triglycerides, reduced high-density lipoprotein cholesterol (HDL-C), hypertension, and elevated fasting glucose—significantly increases the risk of AF. The primary mechanisms through which it contributes to AF include atrial remodeling, autonomic dysfunction, chronic inflammation, oxidative stress, and myocardial fibrosis [7].

## 8. Preclinical Evidence and Animal Models

In order to improve our current treatment options, a deeper insight into the underlying pathophysiological mechanisms of AF is required. Animal models have been instrumental in revealing these pathways and fostering the development of new therapeutic interventions.

Different animal models, ranging from mice to large animals such as horses, and various methods to induce AF, such as rapid atrial pacing, atrial burst pacing, long-term pacing, ischemia, and catheter-based stimulation, have been used to replicate human AF pathophysiology. The wide range of available models underscores the limitations of current approaches. Therefore, careful consideration is required when interpreting animal experimental results and translating findings into clinical practice [142].

Each animal model has its own advantages and disadvantages that influence its suitability for investigating electrical, structural, metabolic, and autonomic remodeling [143]. Rodents are a good option for molecular studies, while canines better model autonomic and structural changes. Goats are suitable for long-term studies of persistent AF. The selection of appropriate model depends on factors such as physiological congruence with humans, the availability of transgenic models, cost, ethical considerations, and experimental feasibility [142, 143].

Animal studies have significantly advanced our understanding of metabolic alterations in AF, with mice being particularly valuable due to their suitability for modeling AF-related comorbidities and their ease of genetic manipulation [143] (Table 1, Ref. [54, 55, 57, 85, 144, 145, 146]). Mouse models have been especially useful in elucidating the role of AMPK signaling in AF-associated metabolic remodeling, as highlighted in earlier sections [55, 56, 57, 58, 143], emphasizing their utility in uncovering molecular pathways that affect atrial metabolism.

**Table 1.** **Selected clinical and preclinical studies assessing metabolic remodeling and mitochondrial stress in atrial fibrillation**.

Species	Method/Animal model	Major results	Ref.
Mice	Atrium-selective genetic depletion of AMPK	AMPK maintains atrial homeostasis and protects against adverse remodeling by modulating transcription factors that regulate ion channels and gap junction proteins.	[54]
Mice	Cardiac-specific LKB1 knockout	LKB1 knockout mice develop spontaneous and persistent AF, mimicking human AF pathology via progressive inflammatory atrial cardiomyopathy and associated electrical and structural remodeling.	[55]
Mice	Murine HFpEF model induced in male mice via high-fat diet and nitric oxide synthase inhibition. Pacing-induced AF	Metformin, an AMPK activator, reduced AF vulnerability in these mice, suggesting that impaired AMPK signaling contributes to electrophysiological instability in the context of metabolic disorders.	[57]
Gout	Goat atrial tissue was sampled at sinus rhythm and at 1, 2, 4, 8, and 16 weeks of burst pacing–induced AF	Sustained AF caused a 60% drop in atrial phosphocreatine levels, which normalized by 8–16 weeks, indicating metabolic adaptation to increased energy demands.	[145]
Sheep	Direct short electrical stimulation of the right atrium to induce AF	After 2 hours of pacing, mitochondrial FoF_1_-ATPase activity increased, while cytochrome c oxidase and Na^+^/K^+^-ATPase α_1_-subunit expression and activity remained unchanged.	[146]
Human	Samples of left atrial myocardium of patients with AF compared to matched samples of patients with sinus rhythm	Increased CaMKII and AMPK expression, along with elevated FAT/CD36 at the membrane, led to lipid accumulation, reduced GLUT-4 membrane expression, increased glycogen storage, and higher pro-apoptotic bax levels.	[144]
Human	Samples of right atrial tissue	MtDNA deletions associated with aging or AF can impair ATP synthesis, leading to bioenergetic deficiency in the human atrium.	[85]

AMPK, AMP-activated protein kinase; LKB1, liver kinase B1; AF, atrial fibrillation; HFpEF, heart failure with preserved ejection fraction; FoF_1_-ATPase, adenosine triphosphate synthase; CaMKII, Ca^2+^/calmodulin-dependent protein kinase II; GLUT-4, glucose transporter-4; mtDNA, mitochondrial DNA; ATP, adenosine triphosphate; FAT/CD36, fatty acid translocase/cluster of differentiation 36.

Large animal models have also underscored metabolic mechanisms in AF. In a goat model, long-lasting AF caused a 60% reduction in phosphocreatine levels, which returned to baseline within 8–16 weeks, indicating metabolic adaptation to increased energy demands and supporting the “relative ischemia” hypothesis in AF [145]. Similarly, in a sheep model of pacing-induced AF, mitochondrial adenosine triphosphate synthase (FoF_1_-ATPase) activity increased after just 2 hours of pacing, while cytochrome c oxidase activity and Na^+^/K^+^-ATPase α_1_-subunit expression and activity remained unchanged [146] (Table 1).

Overall, animal models have revealed fundamental aspects of metabolic remodeling and mitochondrial dysfunction in AF. However, they often fall short of accurately replicating the physiology of the human heart. *In vitro* models using human cardiomyocytes from patients undergoing heart surgery appear to address this limitation [147]. These models allow detailed microscopic analysis, can be genetically modified, and are suitable for atrial-selective antiarrhythmic drug screening. Human pluripotent stem cells (hPSCs) are the primary source of cardiomyocytes for such *in vitro* models [147].

Samples of left atrial myocardium from individuals with AF showed dysregulated metabolism compared to those in sinus rhythm, including elevated expression of CaMKII and AMPK, increased membrane levels of fatty acid translocase/cluster of differentiation 36 (FAT/CD36), lipid accumulation, reduced GLUT-4 membrane expression, increased glycogen storage, and higher levels of the pro-apoptotic protein Bax [144]. In addition, analysis of postoperative atrial tissue samples from AF patients revealed mtDNA deletions that impair ATP synthesis [85].

The heterogeneity of AF duration and etiology complicates the standardization of a metabolism-specific experimental model. Human studies typically focus on valvular or permanent AF to reduce the effect of confounding factors, whereas animal models often employ electrical overstimulation to induce AF. As a result, although some aspects of metabolic remodeling appear to be consistently observed across human and animal studies, others are related to specific experimental conditions.

## 9. Clinical Correlates and Human Studies

### 9.1 Atrial Genomic, Metabolomic and Transcriptomics Analyses in AF Patients

Integration of genome-to-metabolome datasets—spanning genomics, transcriptomics, epigenomics, proteomics, and metabolomics—has sharpened mechanistic resolution of metabolic stress in AF. Cross-omic synthesis within a systems framework is uncovering tractable therapeutic nodes and enabling biomarker-driven patient stratification [148, 149, 150].

In parallel, genome-wide association studies (GWAS) remain foundational for charting common-variant architecture by contrasting allele-frequency distributions—most commonly single-nucleotide polymorphisms (SNPs)—between cases and controls. A meta-analysis of 33 GWAS, including 22,346 patients with AF and 132,086 referents, identified variants in the paired like homeodomain 2 (*PITX2*) genomic region as the only ones significantly associated with AF across European, African, and Japanese ancestries [151]. In animal *PITX2* knockout models, atrial enlargement was associated with abnormalities in cellular ultrastructure, which included disrupted intercalated discs and swollen mitochondria in atrial cardiomyocytes [152]. Moreover, in a *PITX2* knockout zebrafish model, indications of atrial fibrosis and enlargement, metabolic changes, and oxidative stress were observed prior to the initiation of AF [153]. In the human heart, *PITX2* deficiency has been associated with atrial mitochondrial dysfunction and a metabolic shift to glycolysis. These metabolic alterations may be responsible for the structural and functional abnormalities observed in *PITX2*-deficient atria [154].

In a study involving human atrial tissues from 10 patients with non-valvular AF compared to 10 healthy donors, Liu *et al*. [155] uncovered some significant insights into metabolic reprogramming using a combined transcriptomic and proteomic approach. Their metabolomics analysis revealed 113 metabolites that were upregulated and 10 that were downregulated, with enrichment of pathways linked to mitochondrial energy metabolism. On the proteomic side, the investigators found 330 proteins that were expressed differently (225 upregulated and 105 downregulated), among which glycerol-3-phosphate dehydrogenase 2 (GPD2), plectin (PLEC), and synemin (SYNM) were recognized as key hub proteins. Gene Set Variation Analysis (GSVA) showed significant changes in mitochondrial pathways, particularly in oxidative phosphorylation and ATP biosynthesis, among AF patients. These findings indicate that impaired mitochondrial metabolism is a crucial factor in AF pathogenesis [155]. Furthermore, quantitative acetylated proteomics analysis has been used to assess acetylation changes in left atrial tissues from 18 patients (9 with chronic AF and 9 with sinus rhythm). The investigators identified 352 differentially acetylated sites across 193 proteins, most of which are involved in regulating atrial metabolism and contraction. Interestingly, most acetylation sites related to energy metabolism were found to be increased in AF, while those associated with muscle contraction were decreased. These changes indicate that acetylation is a crucial factor in the pathological processes that cause metabolic and contractile remodeling, and could lead to new therapeutic targets [156].

In another study, Barth *et al*. [33] used Affymetrix U133 arrays to examine ventricular gene profiles and atrial mRNA expression in 10 patients with permanent AF compared to 20 with sinus rhythm. The investigators identified 1434 genes that were deregulated in AF-related atrial tissue, mostly downregulated, including key Ca^2+^ signaling components. Functional classification based on Gene Ontology indicated changes related to structural and electrophysiological remodeling. The observed upregulation of metabolic transcripts suggested an adaptive response to meet increased energy demand. Of note, the fibrillating atrium exhibited dedifferentiation with adoption of a ventricular-like gene expression pattern, which augmented glucose metabolism and reduced fatty acid oxidation [33].

Lastly, combined metabolomic and proteomic analysis of cardiac tissue from patients with persistent AF showed elevated levels of β-hydroxybutyrate, the primary substrate in ketone body metabolism, as well as increased levels of ketogenic amino acids and glycine. This study was conducted using high-resolution proton nuclear magnetic resonance (NMR) spectroscopy [157].

### 9.2 Imaging of Myocardial Energetics

Clarifying the pathophysiological basis and prognostic weight of metabolic remodeling in atrial fibrillation requires robust clinical indicators or surrogate biomarkers that reflect these shifts. Although echocardiography and cardiac computed tomography (CT) cannot directly visualize metabolic derangements in the fibrillating atria, they capture their structural and functional sequelae—most notably atrial enlargement and impaired contractility [158]. By contrast, molecular imaging platforms such as positron emission tomography (PET), single-photon emission computed tomography (SPECT), and magnetic resonance spectroscopy (MRS) offer noninvasive interrogation of myocardial energy metabolism and substrate use, and thus hold considerable promise for phenotyping the metabolic substrate [159].

^18^F-fluorodeoxyglucose (FDG) PET has gained increasing interest owing to its intrinsic quantitative capabilities in functional and molecular cardiac imaging. Under fasting conditions, FDG uptake reflects a range of pathological processes such as ischemia, inflammation, and pressure overload [160]. Recent advances in digital PET technology have improved our ability to measure FDG uptake in the thin wall of the left atrium (LA) [161]. Many studies have highlighted increased FDG uptake in the atria of AF patients, suggesting higher local metabolic activity and inflammation [162, 163, 164, 165]. Interestingly, higher FDG uptake in the right atrium (RA) has been linked to increased levels of B-type natriuretic peptide (BNP) and may help predict the success of AF termination after radiofrequency catheter ablation [163]. Moreover, a recent study using nicotinic acid-stimulated PET showed that FDG uptake in the LA was significantly higher in patients with persistent AF compared to healthy individuals. This uptake decreased after normal heart rhythm was restored [166].

SPECT offers intrinsic benefits such as high sensitivity and wide availability and can assess myocardial function. However, its use in clinical settings is limited due to technical challenges. These include complex radiotracer metabolism, low spatial and temporal resolution, and inadequate photon attenuation correction. These limitations hinder its ability to quantify cellular metabolic activity in the thin and small atrial walls [159]. As a result, the role of SPECT in diagnosing atrial metabolic stress in AF is still uncertain.

MRS is a noninvasive method that does not involve radiation and detects metabolic signals from nuclei such as ^31^P, ^1^H, and ^23^Na [167]. When combined with cardiac magnetic resonance imaging (MRI), it provides a detailed view of cardiac metabolism, structure, and function. Although ^31^P-MRS has been successfully applied to evaluate left ventricular (LV) metabolism, its use in the atria is limited by low reproducibility, poor resolution, and long acquisition times [167, 168]. Additionally, the LV PCr/ATP ratio measured via ^31^P-MRS does not correlate with mitochondrial respiratory capacity in RA appendage tissue, highlighting the need for further research [169].

### 9.3 Biomarkers

Efforts to refine risk assessment in AF increasingly center on blood-based markers. Natriuretic peptides (BNP, N-terminal pro–B-type natriuretic peptide (NT-proBNP), atrial natriuretic peptide (ANP)), cardiac troponin T, suppression of tumorigenicity 2 (ST2), tissue inhibitor of metalloproteinases 1 (TIMP1), insulin-like growth factor 1 (IGF1)/insulin-like growth factor–binding protein 1 (IGFBP1), endothelial adhesion molecules (intercellular adhesion molecule 1 (ICAM1), vascular cell adhesion molecule 1 (VCAM1)), inflammatory chemokines such as C-C motif chemokine ligand 2 (CCL2), and protease-activated receptors (PAR1–PAR4) have been interrogated predominantly as predictors of AF onset and progression; comparatively fewer investigations address their value for severity stratification [170, 171, 172, 173, 174, 175]. Modulators of mitochondrial dysfunction, such as circulating cell-free mitochondrial DNA (cfc-mtDNA), 8-hydroxy2^′^-deoxyguanosine (8-OHdG), and heat shock proteins (HSPs), may help in staging the severity of AF and assessing treatment outcomes [176].

Cfc-mtDNA enters the bloodstream through cell necrosis or active secretion [177]. It is successfully used as a biomarker for conditions associated with mitochondrial stress, such as cardiovascular disease and cancer progression [178, 179]. Cfc-mtDNA has been identified as a putative atrial-specific biomarker, released into the bloodstream in association with the structural and metabolic remodeling that accompanies AF progression. Findings from an observational study in subjects with different phenotypes of AF revealed significant associations between plasma cfc-mtDNA concentrations and both AF stage, especially paroxysmal AF, and recurrence of AF following AF treatment [95]. Interestingly, cfc-mtDNA levels were significantly lower in AF patients with tachycardia-induced cardiomyopathy (TIC) compared to non-TIC AF patients, suggesting it may serve as a potential biomarker for predicting TIC in individuals with AF [180].

8-OHdG is a marker of oxidative DNA damage and has been identified as a potential serum biomarker for AF [176, 181]. Both paroxysmal and chronic AF patients have been observed to have higher levels of 8-OHdG. These levels correlate with the stage and severity of the disease [91, 182]. Additionally, levels rise in patients who develop postoperative AF and decrease after ablation therapy, indicating both diagnostic and prognostic value [183, 184]. These findings highlight the potential of blood-based markers like 8-OHdG to reflect the molecular processes underlying the development of AF [176].

Lastly, clinical research has not consistently demonstrated a link between plasma HSP levels and AF stage or recurrence, despite the fact that HSPs, including HSP60 and HSP10, have shown cardioprotective benefits in experimental AF models [91, 185].

## 10. Therapeutic Targets: Current and Emerging

As mentioned above, mitochondrial dysfunction and oxidative stress promote structural, metabolic, and electrical alterations in the atria, leading to increased susceptibility to AF. Several widely prescribed pharmacological agents, including angiotensin-converting enzyme (ACE) inhibitors, statins, carvedilol and ranolazine, along with nutraceutical compounds such as coenzyme Q10 (CoQ10), N-acetylcysteine, and L-glutamine, have been shown to exert indirect modulatory effects on mitochondrial function and may represent potential therapeutic strategies for the management of AF [122]. Nevertheless, future studies are needed towards this direction.

Therapeutic modulation of mitochondrial dysfunction—including mitochondria-targeted antioxidants, membrane-stabilizing compounds, and agents that augment mitochondrial biogenesis—remains under active study but is still predominantly preclinical, with notable translational headwinds (Table 2, Ref. [40, 57, 186, 187, 188, 189, 190, 191, 192, 193, 194, 195]). Elamipretide, a mitochondria-directed tetrapeptide, stabilizes cardiolipin and tightens ETC coupling; although clinical benefit has been shown in heart failure cohorts, antiarrhythmic efficacy in AF has yet to be established [196]. MitoQ, a mitochondria-targeted antioxidant, has gained attention for its cardioprotective properties. Treatment with MitoQ ameliorates aortic stiffness in old mice and improves endothelial function. Additionally, it may counteract atrial remodeling by mitigating ROS within mitochondria [186]. Trimetazidine functions as a metabolic modulator, optimizing complex I activity and engaging transcriptional programs regulating mitochondrial biogenesis. It has been proposed that trimetazidine may possess anti-AF properties [40]. Indeed, using a canine model of heart failure, Li *et al*. [187] demonstrated that trimetazidine attenuates tachycardia-induced atrial ultrastructural remodeling, reduces AF inducibility, and shortens AF duration. Perhexiline, another metabolic modulator, shifts myocardial energy metabolism from fatty acid oxidation toward increased carbohydrate utilization, thereby preserving ATP levels while reducing O_2_ consumption—an action that may counteract metabolic remodeling associated with AF [188]. Finally, KL1333, a novel NAD^+^ modulator, may exert antiarrhythmic effects through activation of the AMPK/SIRT1/PGC-1α axis, a central regulator of mitochondrial bioenergetics and resilience [189].

**Table 2.** **Investigational and emerging agents targeting metabolic remodeling and mitochondrial dysfunction in atrial fibrillation**.

Agent		Primary target/pathway	Proposed mechanism(s) of action	Key effects/evidence
Mitochondria-targeted antioxidants				
	MitoQ	Mitochondria	Mitochondria-directed antioxidant; attenuates mitochondrial ROS and preserves organellar function.	In aged mice, it reduces aortic stiffness and improves endothelial function; may limit atrial remodeling via ROS neutralization [186].
	Trimetazidine	Mitochondria	Metabolic modulation with optimization of complex I activity; engages mitochondrial biogenesis programs (PPARs/PGC-1α); lowers oxidative stress.	Prevents atrial structural remodeling, reduces AF inducibility, and shortens AF duration [40, 187].
	Perhexiline	Mitochondria	Reprograms substrate utilization from fatty-acid oxidation to greater carbohydrate use; preserves ATP and reduces O_2_ consumption.	Prevents metabolic remodeling associated with AF [188].
	KL1333	Mitochondria	NAD^+^ modulation with activation of the AMPK/SIRT1/PGC-1α axis.	Developmental stage: under investigation [189].
Glucose-lowering drugs				
	SGLT2 inhibitors	SGLT2	Anti-inflammatory, antifibrotic, antioxidant, and electrophysiologic effects; improve mitochondrial function, Ca^2+^ handling, and metabolic efficiency.	Dapagliflozin reduced AF and atrial flutter (AFL) events by 19%, irrespective of baseline AF status [190]. Large meta-analyses have shown that SGLT2is significantly reduce the risk of AF in patients with diabetes and HF [190, 191, 192].
	GLP-1 RAs	Glucagon-like peptide-1 receptor	Antifibrotic, anti-inflammatory, antioxidant, and anti-apoptotic actions; metabolic reprogramming with improved mitochondrial function.	Meta-analysis involving patients at high cardiovascular risk showed that semaglutide significantly reduced the incidence of AF by 42% [193].
	Metformin	Gluconeogenesis	AMPK-driven metabolic and electrophysiological modulation.	Metformin-treated mice showed significantly increased AMPK signaling, which was associated with reduction in AF susceptibility, as evidenced by significantly lower AF inducibility and shorter duration compared to the placebo group [57].
Anticoagulants				
	Rivaroxaban	Mitochondria, anticoagulation pathway	Inhibits FXa, enhances mitophagy, improves mitochondrial membrane potential, reduces ROS production, increases the enzymatic activity of citrate synthase and cytochrome C oxidase.	Enhanced mitochondrial function in HCAECs exposed to high glucose [194].
	Edoxaban	Mitochondria, anticoagulation pathway	Inhibits FXa, enhances mitochondrial oxygen consumption during maximal oxidative phosphorylation, increases ATP production.	Prevented factor Xa-induced mitochondrial impairment in the human lung carcinoma cell line A549 [195].

ROS, reactive oxygen species; NAD^+^, nicotinamide adenine dinucleotide; AMPK, AMP-activated protein kinase; SIRT1, sirtuin 1; PPARs, peroxisome proliferator-activated receptors; PGC-1α, peroxisome proliferator-activated receptor-gamma coactivator-1alpha; SGLT2, sodium-glucose cotransporter 2; GLP-1 RAs, glucagon-like peptide-1 receptor agonists; AF, atrial fibrillation; HF, heart failure; FXa, activated factor X; HCAECs, human coronary artery endothelial cells; ATP, adenosine triphosphate.

Sodium–glucose cotransporter-2 inhibitors (SGLT2is) are a modern class of oral glucose-lowering agents that selectively block proximal tubular SGLT2, reducing renal reabsorption of filtered glucose and sodium. The resulting glycosuria and natriuresis support glycemic control and confer favorable hemodynamic effects. Beyond glycemic control, SGLT2is have demonstrated distinct cardio–renal–metabolic benefits and, owing to their multifactorial pathophysiological mechanisms, have emerged as promising therapeutic agents in the pharmacological management of AF [197, 198, 199, 200] (Table 2).

A post hoc analysis of the DECLARE-TIMI 58 trial showed that dapagliflozin reduced AF and atrial flutter (AFL) events by 19% (7.8 vs. 9.6 events per 1000 patient-years; HR 0.81, 95% CI: 0.68–0.95, *p* = 0.009) over 50.4 months, irrespective of baseline AF status [190]. According to a relative recent meta-analysis addressing the effect of SGLT2is on cardiac arrhythmias in patients with heart failure, diabetes mellitus (DM), and chronic kidney disease, which included 22 clinical trials and more than 52,000 patients, it was found that gliflozins had significantly reduced the risk of AF (RR: 0.82; 95% CI: 0.70–0.96) and embolic stroke (RR: 0.32; 95% CI: 0.12–0.85), independent of baseline glycemic status [191]. Similar were the findings of another published meta-analysis of 22 trials involving patients with DM and heart failure in which SGLT2is were associated with an 18% reduction in AF/AFL incidence compared to control group (OR = 0.82, 95% CI: 0.73–0.93, *p* = 0.002) [192].

The anti-arrhythmic mechanisms by which SGLT2is reduce the risk for AF remain unclear. Proposed mechanisms involve their anti-inflammatory properties, including the suppression of circulating pro-inflammatory cytokines such as TNF-α and transforming growth factor-β (TGF-β), and the modulation of NLRP3 inflammasome activity. SGLT2is can also reduce ROS production, ameliorate mitochondrial respiratory capacity and intracellular Ca^2+^ handling, suppress pro-fibrotic signaling pathways, including the TGF-β/SMAD axis, and improve parameters of atrial electrophysiology, thus reducing AF inducibility [201].

Glucagon-like peptide-1 receptor agonists (GLP-1 RAs) are established incretin therapies that achieve glycemic control by potentiating glucose-dependent insulin secretion while restraining inappropriate glucagon release [202]. Of importance, beyond glycemia, cardiovascular outcome trials in type 2 diabetes consistently show reductions in non-fatal myocardial infarction, stroke, and cardiovascular mortality with GLP-1 RAs [203, 204]. However, these studies were neither designed nor powered to assess atrial fibrillation, and the effect of GLP-1 RA therapy on AF risk remains uncertain. Nevertheless, a recently published meta-analysis of ten randomized clinical trials (RCTs) involving patients at high cardiovascular risk showed that semaglutide significantly reduced the incidence of AF by 42% as compared to placebo [193].

Theoretically, GLP-1 RAs may exert antiarrhythmic effects because of their pleotropic effects (Table 2). These agents exert cardiometabolic benefits by facilitating glucose uptake through p38 mitogen-activated protein kinase (MAPK)-mediated translocation of glucose transporters and enhancing mitochondrial fatty acid oxidation. Collectively, these effects alleviate metabolic stress within the myocardium, a key contributor to arrhythmogenic remodeling [205]. GLP-1 RAs have also shown efficacy in modulating antioxidant and anti-apoptotic responses, preserving mitochondrial function, and reducing cardiac hypertrophy and fibrosis [206, 207, 208, 209]. These mechanisms highlight the potential anti-AF properties of GLP-1 RAs, warranting further investigation.

Direct oral anticoagulants (DOACs) have gradually replaced vitamin K antagonists (VKAs) as treatments for thromboembolism in patients with AF, due to their comparable efficacy and improved safety profile [210]. DOACs act as direct inhibitors of activated factor X (FXa) or thrombin (FIIa). These coagulation factors exert a number of additional biological actions, including ROS production, regulation of mitochondrial function, and pro-inflammatory and pro-fibrotic responses [211, 212, 213, 214]. Thus, DOACs may preserve mitochondrial function and reduce oxidative stress, potentially offering clinical advantages over VKAs, which have been reported to induce mitochondrial damage in lymphocytes [194, 211, 212, 213, 215, 216] (Table 2).

Gene-directed modulation of mitochondrial homeostasis—targeting master regulators such as PGC-1α, mitochondrial catalase (mCAT), and TFAM—is emerging as a credible path toward clinical translation [40]. Of importance, advances in nucleic-acid delivery, including lipid nanoparticle platforms and adenoviral vectors, offer the means to engage the arrhythmogenic substrate of AF with greater specificity and therapeutic leverage [10].

## 11. Future Directions and Research Gaps

Beyond the well-established concepts of structural and electrical remodeling, accumulating evidence suggests that metabolic remodeling can promote the development of AF through changes in cellular metabolism and energy homeostasis in the atrial myocardium. Mitochondrial stress is also increasingly recognized as a key contributor of this process.

The newly proposed guidelines for the management of AF recognize it as a complex cardiovascular disease that requires a more holistic and individualized management strategy. Concurrently, therapeutic pillars include lifestyle and risk factor modification, anticoagulation, and early rhythm control strategies. This comprehensive approach is particularly important in patients with metabolic diseases such as diabetes and obesity. These disorders contribute directly to the development and progression of atrial cardiomyopathy through diverse and interrelated pathways, making AF more challenging to manage.

Targeting atrial metabolism in individuals with AF represents a novel therapeutic avenue that aims directly on mitochondrial stress and metabolic remodeling. Pharmacological agents such as trimetazidine, perhexiline, and DOACs have shown potential to improve mitochondrial function and reduce oxidative stress. Furthermore, mitochondria-targeted antioxidants and NAD^+^ modulators, as well as gene-based strategies targeting PGC-1α and TFAM, may offer disease-modifying benefits by attenuating atrial metabolic remodeling. Although these approaches appear promising, they are still in the preclinical stages of development and require more rigorous research.

SGLT2is and GLP-1RAs, owing to their multifaceted actions, have emerged as promising agents in the pharmacological management of atrial metabolic remodeling through their direct and predominantly indirect properties to regulate key metabolic pathways. Future research should aim on conducting large-scale RCTs with pre-specified AF endpoints to conclusively establish the efficacy of SGLT2is, GLP-1RAs, and their combination in preventing atrial cardiomyopathy and AF.

Despite meaningful progress, metabolic imaging—most commonly FDG-PET and hyperpolarized MRI—remains constrained in routine practice. Limited spatial resolution, together with variability in acquisition and post-processing, reduces the capacity to resolve early metabolic perturbations within the atrial myocardium. In effect, these technical issues blunt the detection of nascent remodeling and complicate comparisons across studies and centers. Circulating markers of mitochondrial injury, including cell-free mitochondrial DNA and 8-hydroxy-2^′^-deoxyguanosine, offer a noninvasive readout of atrial metabolic status. Their clinical role, however, is not yet settled. Pre-analytical handling, assay calibration, and population-level reproducibility remain incompletely addressed and require prospective, multicenter validation. At the systems level, integrative multi-omics is widening the catalogue of candidate biomarkers and pharmacologically tractable pathways, enabling finer phenotyping and, ultimately, individualized therapy. In parallel, artificial intelligence—particularly deep-learning methods—promises more reliable image interpretation and coherent synthesis of multimodal data, with potential gains in diagnostic precision, risk stratification, and decision-making in AF [195, 217].

Delivering on this promise will require coordinated effort across basic, translational, and clinical domains, with explicit attention to methodological harmonization and external validation.

## 12. Conclusion

AF remains a growing global burden, underpinned by a pathophysiology that extends beyond traditional concepts of electrical and structural remodeling. Metabolic remodeling and mitochondrial stress are increasingly recognized as central components of AF pathophysiology and, at the same time, potential therapeutic targets. Ultimately, embedding the concept of atrial metabolic remodeling into clinical practice will redefine AF management, shifting the focus from arrhythmia suppression to substrate modification. This approach may yield long-lasting improvements in AF-related outcomes and eventually prevent its onset.
